# Bioinformatics integrated analysis to investigate candidate biomarkers and associated metabolites in osteosarcoma

**DOI:** 10.1186/s13018-021-02578-0

**Published:** 2021-07-05

**Authors:** Jun Wang, Mingzhi Gong, Zhenggang Xiong, Yangyang Zhao, Deguo Xing

**Affiliations:** Department of Orthopedics and Trauma, The Second Hospital of Shandong University, No. 247 Beiyuan Street, Jinan, 250033 China

**Keywords:** Osteosarcoma, Prognosis, Biomarkers, Metabolite

## Abstract

**Background:**

This study hoped to explore the potential biomarkers and associated metabolites during osteosarcoma (OS) progression based on bioinformatics integrated analysis.

**Methods:**

Gene expression profiles of GSE28424, including 19 human OS cell lines (OS group) and 4 human normal long bone tissue samples (control group), were downloaded. The differentially expressed genes (DEGs) in OS vs. control were investigated. The enrichment investigation was performed based on DEGs, followed by protein–protein interaction network analysis. Then, the feature genes associated with OS were explored, followed by survival analysis to reveal prognostic genes. The qRT-PCR assay was performed to test the expression of these genes. Finally, the OS-associated metabolites and disease-metabolic network were further investigated.

**Results:**

Totally, 357 DEGs were revealed between the OS vs. control groups. These DEGs, such as CXCL12, were mainly involved in functions like leukocyte migration. Then, totally, 38 feature genes were explored, of which 8 genes showed significant associations with the survival of patients. High expression of CXCL12, CEBPA, SPARCL1, CAT, TUBA1A, and ALDH1A1 was associated with longer survival time, while high expression of CFLAR and STC2 was associated with poor survival. Finally, a disease-metabolic network was constructed with 25 nodes including two disease-associated metabolites cyclophosphamide and bisphenol A (BPA). BPA showed interactions with multiple prognosis-related genes, such as CXCL12 and STC2.

**Conclusion:**

We identified 8 prognosis-related genes in OS. CXCL12 might participate in OS progression via leukocyte migration function. BPA might be an important metabolite interacting with multiple prognosis-related genes.

**Supplementary Information:**

The online version contains supplementary material available at 10.1186/s13018-021-02578-0.

## Highlights

1. We identified 8 prognosis-related genes in OS.
2. CXCL12 might take part in OS via leukocyte migration function.
3. CXCL12 and STC2 might be used as novel biomarkers for OS.
4. Bisphenol A might be an important metabolite interacting with multiple genes.

## Background

Osteosarcoma (OS) is one of the most common malignant bone tumors in children and adolescents under 20 years old [1], accounting for about 5% of all children’s tumors [2]. Current optimal treatment for OS consists of multi-agent chemotherapy and aggressive surgical resection of all sites of disease involvement [3]. Unfortunately, in patients with recurrent or metastatic OS, the long-term survival rate is only 20% [4]. Thus, it is important to further investigate the detailed pathological mechanism of OS.

The mutation of certain genes is responsible for syndromes that predispose to OS [5]. It has been proved that some genes are differentially expressed between OS samples and normal samples in humans, which can be further used for the prediction of chemotherapy response [6]. A previous study shows that the differentially expressed genes (DEGs) such as SRY-Box transcription factor 2 are taking part in the development of OS via influencing cell stemness and migration [7]. A recent study indicates that cyclin E1 is overexpressed in OS samples, which can be used as a prognostic biomarker and potential therapeutic target in OS [8]. Dong et al. proved that the lung adenocarcinoma transcript 1 that related with metastasis promoted the proliferation and metastasis of OS cells by stimulating the PI3K/Akt pathway [9]. Actually, the emerging biomarkers in OS have been revealed not only from genomics but also via metabolomics [10]. A previous study shows that catecholamines and their receptors can be potential molecular markers for OS [11]. It has been proved that metabolites such as parathyroid hormone peptides through the regulation of hyaluronan metabolism affect OS cell migration [12]. A previous global microarray analysis by Namløs et al. [13] revealed that several microRNA (miRNA)-gene interactions (such as miR-9/TGFBR2) were implicated in the development of OS. However, the potential biomarkers and their associated detail molecular mechanism during the OS progression is unknown.

Based on the microarray data provided by Namløs et al. [13], the current bioinformatics analysis was performed to reveal potential DEGs between human OS cell lines and human normal long bone tissue samples, followed by the function and pathway enrichment analysis based on these DEGs. The feature genes for OS were explored based on online databases, followed by the survival analysis to reveal prognostic genes. Finally, the OS-associated metabolites and networks were further investigated. Figure S1 shows the workflow of this study. We hoped to explore the potential biomarkers and associated molecular mechanisms during OS progression.

## Material and methods

### Data resource and preprocessing

A gene expression profile GSE28424 including 19 human OS cell lines (OS group) and 4 human normal long bone tissue samples (control group) was obtained from GEO database (platform: GPL570: Illumina HumanWG-6 v2.0 expression beadchip). As described in the study of Namløs et al. [13], the 19 human OS cell lines, HAL, HOS, 143B, IOR/MOS, IOR/OS9, IOR/OS10, IOR/OS14, IOR/OS15, IOR/OS18, SARG, KPD, MG-63, MHM, MNNG/HOS, OHS, OSA, Saos-2, U-2 OS, and ZK-58 were derived from ATCC or different partner laboratories within EuroBoNeT. Cell line authentication was performed by STR DNA profiling using Powerplex 16. The four normal long bone tissue samples were obtained from amputations of cancer patients at the Norwegian Radium Hospital. The processed gene expression matrix file was obtained using Affy package in R software [14]. Probes with different gene symbols were excluded from this study, and the average value of genes matched to multiple probes was taken as the expression value of the probe.

### Differentially expression analysis

The limma package (version: 3.34.9) of R [15] was used to investigate DEGs between two groups based on the linear regression and empirical Bayesian methods. DEGs were screened by Benjamini & Hochberg (BH) adjusted P < 0.05 and |log_2_ fold change (FC)| > 2. Then, the results were visualized by volcano plots and clustering heatmap.

### Functional enrichment analysis of DEGs

Gene ontology-biological process (GO-BP) function and The Kyoto Encyclopedia of Genes and Genomes (KEGG) pathway enrichment analyses of DEGs were performed using the clusterProfiler package (version: 3.2.11) in R software [16] with thresholds of P value < 0.05 and count ≥ 1.

### The difference analysis of KEGG between OS group and control group

Based on the enrichment background (c2.cp.kegg.v7.2.symbols.gmt) in MSigDB v7.2 database [17], the enrichment scores of each KEGG in each sample of GSE28424 were calculated to obtain a scoring matrix using gene set variation analysis (GSVA) algorithm in R package. Then, the differential expression analysis between the OS group and control group was performed on each KEGG item using the limma package in R software. Finally, the adjusted P < 0.05 was considered as the cut-off value for significantly different items.

### Protein–protein interaction (PPI) network

The Search Tool for the Retrieval of Interacting Genes (STRING) database (version: 11) [18] provides experimental and predicted interaction information. The hub-proteins associated with DEGs were selected according to the STRING database. Then PPI network was constructed by Cytoscape software (version: 3.6.1) [19] with medium confidence (score) = 0.7. The degree (number of the connections for the target protein) was used to evaluate the importance of the target gene.

### The feature gene prediction of OS

The relationship between disease (keyword: osteoporosis)-associated chemical molecular and target gene was revealed by Comparative Toxicogenomics Database (CTD) [20]. Then, based on VENN plot analysis, the screened genes in CTD and genes in GeneCards database [21] were intersected with nodes in the PPI network to explore the feature genes for OS.

### Survival analysis for feature genes

Survival analysis was used to identify biomarkers from significant feature genes. The log_2_(fpkm+1) expression data and clinical information of OS in the TARGET database were downloaded from the University of California Santa Cruz (UCSC) Genome Browser database (https://xenabrowser.net/datapages/?cohort=GDC%20TARGET-OS&removeHub=https%3A%2%2Fxena.treehouse.gi.ucsc.edu%3A443) [22]. The results were visualized using Kaplan-Meier plots. The samples were divided into two groups (high and low) based on median expression level, followed by the overall survival computed between OS and normal by K-M survival curve.

### Real-time reverse transcription PCR (qRT-PCR)

Human OS cell line 143B was obtained from ATCC and was cultured in DMEM (Gibco) supplemented with 10% heat-inactivated fetal bovine serum (FBS). MC3T3, a murine pre-osteoblast cell line, was acquired from the Shandong Provincial Key Laboratory of Oral Tissue Regeneration. The cells were cultured in α-minimal essential medium (α-MEM) supplemented with 10% heat-inactivated FBS. The expression of all 8 prognostic genes in 143B cells and MC3T3 cells was detected by qRT-PCR. Briefly, total RNAs were extracted using TRizol reagent (cwbiotech, # CW0581) and reverse transcripted using HiFiScript cDNA Synthesis Kit (cwbiotech, # CW2569). The current assay was performed on ABI7900FAST (Thermo Fisher Scientific), and the primers were listed in Table 1. The PCR program was performed with thermocycling conditions: 50°C for 3 min, 95°C for 3 min, 40 cycles of 95°C for 10 s; 60°C for 30s, and melt curve 60 to 95°C (increment 0.5°C for 10s). The 2^-ΔΔCt^ method was used for the investigation of gene expression.

**Table 1:** Amplified sequences of genes and their primers

Primer	Sequence
β-actin	Forward: 5′-AGACCTGTACGCCAACACAG-3′ Reverse: 5′-CGGACTCGTCATACTCCTGC-3′
CXCL12	Forward: 5′-CTACAGATGCCCATGCCGAT-3′ Reverse: 5′-CAGCCGGGCTACAATCTGAA-3′
CEBPA	Forward: 5′-AGAACAGCAACGAGTACCGG-3′ Reverse: 5′-G GCGGTCATTGTCACTGGTCA-3′
SPARCL1	Forward: 5′-ATGGCGATGATGATGGCGAT-3′ Reverse: 5′-GATTGAGCTCTCTCGGCCTC-3′
CFLAR	Forward: 5′-TTGTGCCGGGATGTTGCTAT-3′ Reverse: 5′-AGAGCAGTTCAGCCAAGTCC-3′
CAT	Forward: 5′-AGTGATCGGGGGATTCCAGA-3′ Reverse: 5′-AAGTCTCGCCGCATCTTCAA-3′
STC2	Forward: 5′-CACTGTTTGGTCAACGCTGG-3′ Reverse: 5′-AGCGTGGGCCTTACATTTCA-3′
TUBA1A	Forward: 5′-CTATCCCCGCATCCACTTCC-3′ Reverse: 5′-TTTACCATGGCGAGGGTCAC-3′
ALDH1A1	Forward: 5′-ATCCTCTGACCCCAGGAGTC-3′ Reverse: 5′-AACACTGTGGGCTGGACAAA-3′

### Predictive analysis of metabolites related to OS

The relationship between disease (keyword: osteoporosis)-associated chemical molecular and target gene was revealed by CDT, followed by the combination of prognosis-related genes obtained above to explore the key genes and compounds of OS. The chemicals associated with OS were mapped to metabolite ID with the application of the Compound ID Conversion tool in MetaboAnalyst database (www.metaboanalyst.ca/).

### Disease-metabolic network construction

KEGG pathway graph of DEGs was regarded as the analytic target to construct the differential pathway network that contained chemical compounds, reactions, enzymes, pathways, and KEGG modules using a heat diffusion model-based algorithm [23]. Specifically, the metabolic perturbation can be considered as the heat flow that traverses the KEGG graph. The null diffusive process highlighted that heat could flow out from nodes, which corresponded to the affected metabolites and across the whole differential pathway network. The p values of nodes were calculated according to the diffusion scores and ranked. The formula for the temperature diffusion score was as follows: T = - KI * G.

G indicator is input metabolite, it is 1 if the affected metabolite is entered; otherwise, 0. KI is the conductance matrix and equals to L plus B (L: the unnormalized graph Laplacian and B: the diagonal adjacency matrix). Notably, B (i, i) = 1 if node i is a pathway; otherwise, B (i, i) = 0. Herein, the nodes with p < 0.05 were retained and used to construct the disease-metabolic network.

## Results

### DEGs between the OS group and control group

**Fig. 1:**
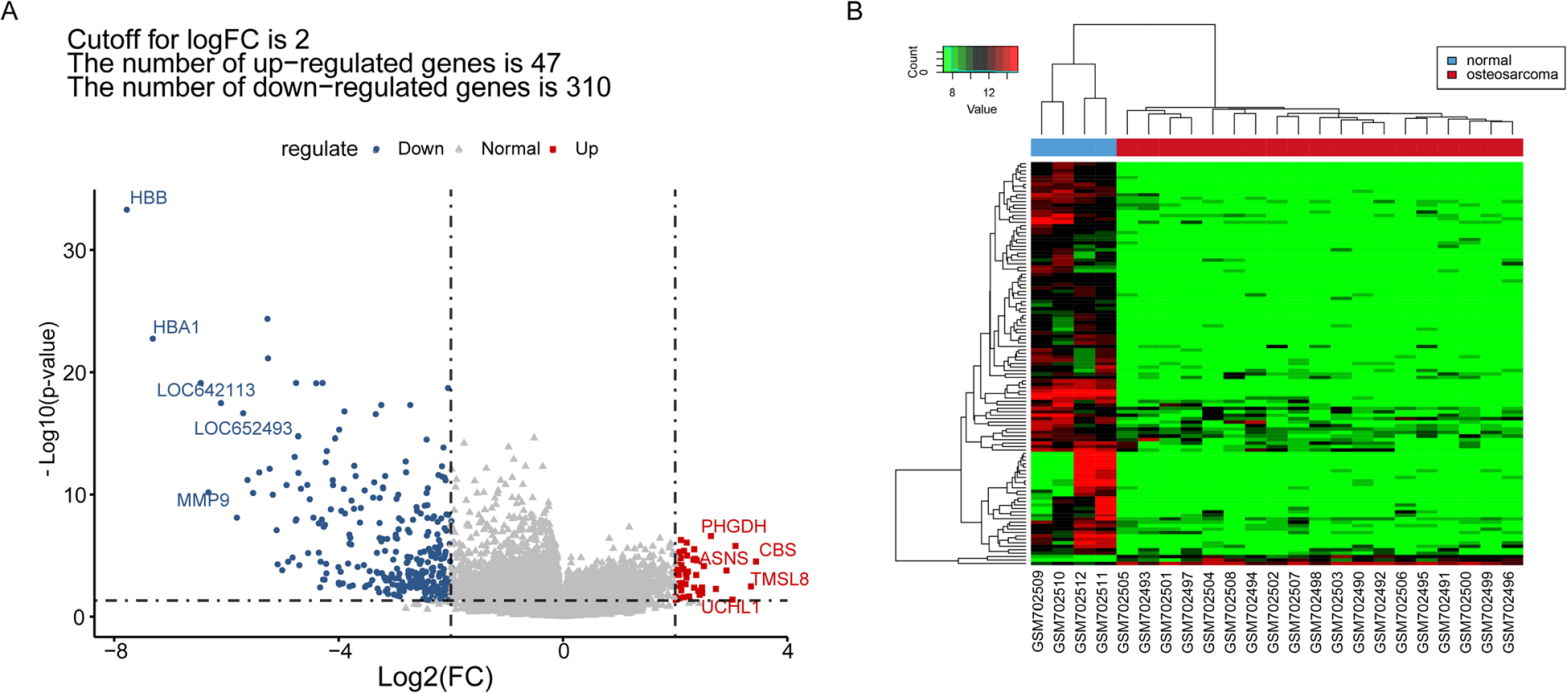
The volcano plots and heatmap for differentially expressed mRNAs between osteosarcoma sample and normal sample. A The volcano plots in current study; the X-axis represented the value of log_2_ fold change, while the Y-axis represented the value of −log10; the red node represented upregulated genes, while the blue node represented the downregulated gene. **B** The heatmap in current study; the red block represented osteosarcoma samples, while green block represented normal samples

A total of 357 DEGs were revealed between the OS group and control group, containing 47 upregulated and 310 downregulated genes. The volcano plot showed that the upregulated genes and downregulated genes were significantly separated (Fig. 1A, B), suggesting a reliable result.

### Enrichment analysis and GSVA investigation

**Fig. 2:**
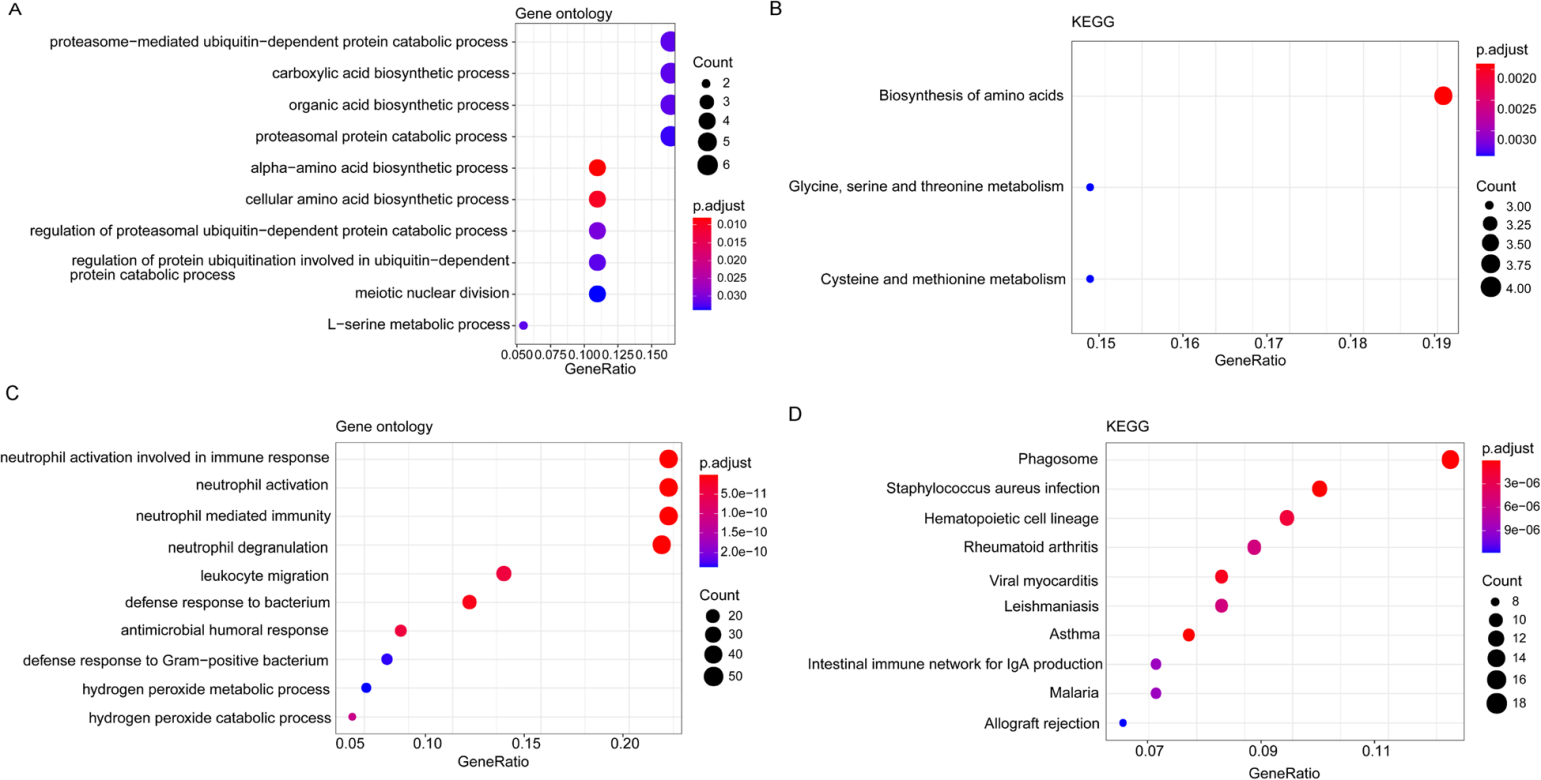
The GO/KEGG pathway enrichment cluster interaction analysis of the differentially expressed mRNAs. **A** The GO functions assembled by upregulated mRNAs. **B** The KEGG pathways enriched by upregulated mRNAs. **C** The GO functions assembled by downregulated mRNAs. **D** The KEGG pathways enriched by downregulated mRNAs. X-axis represented the gene ratio (−log10); Y-axis represented the different items of functions or pathways. The deeper the red, the more significant the p value. The bigger the node, the more number the genes enriched in item

**Fig. 3:**
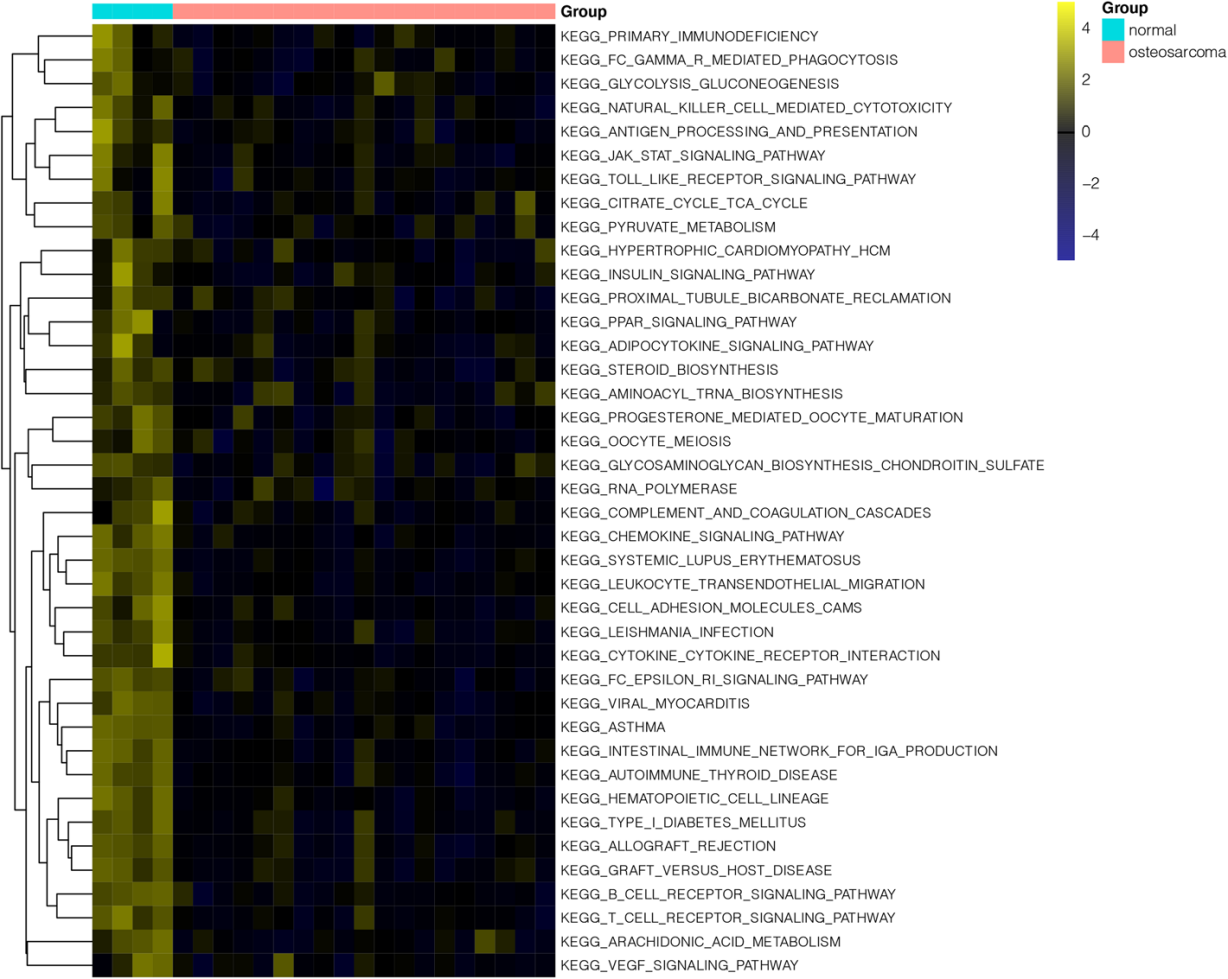
The heatmap of gene set variation analysis for KEGG pathways between osteosarcoma samples and normal samples. The green bar on the top represented samples in normal group, while the red bar on the top represented samples in osteosarcoma group. The color from yellow to black indicated high to low representation value

The upregulated DEGs mainly enriched in 14 GO-BP functions including leukocyte migration (GO:0050900, Genes: C–X–C motif chemokine ligand 12 (CXCL12), etc.) (Fig. 2A) and 3 KEGG pathways such as biosynthesis of amino acids (hsa01230, genes: phosphoglycerate dehydrogenase (PHGDH), etc.) (Fig. 2B). Meanwhile, downregulated DEGs were mainly involved in GO-BP functions including neutrophil activation (GO:0042119, genes: Fc fragment of IgG receptor IIIb (FCGR3B), etc.) and pathways like phagosome (hsa04145, genes: FCGR3B, etc.) (Fig. 2C, D).

Furthermore, the GSVA analysis on the KEGG pathway revealed that a totally 40 outstanding pathways showed the difference between OS and normal samples, such as vascular endothelial growth factor (VEGF) signaling pathway, cell adhesion molecules, and chemokine signaling pathway (Fig. 3).

### Feature gene investigation and survival analysis

**Fig. 4:**
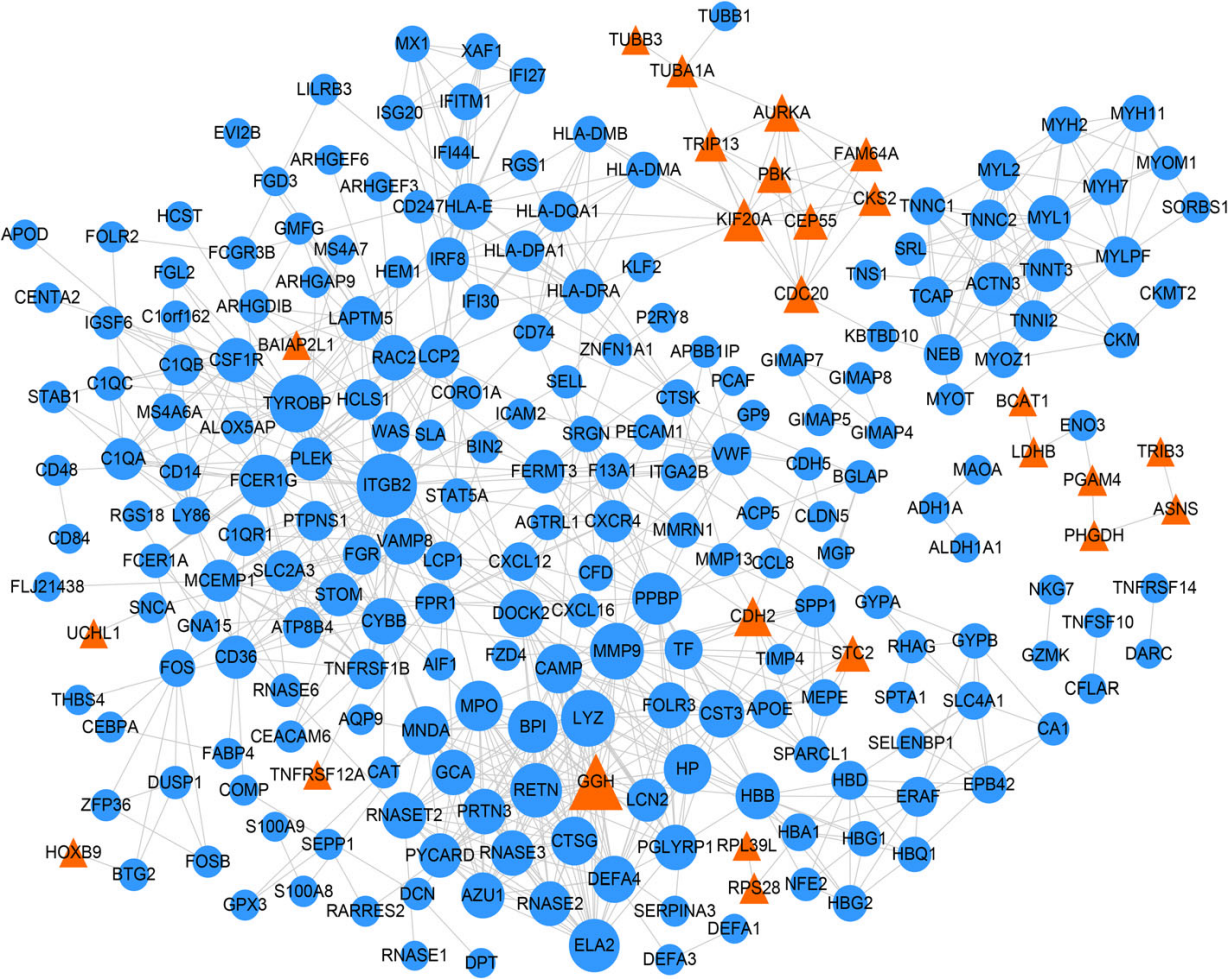
Protein–protein interaction network in current study. The blue circle represented downregulated gene, while the orange triangle represented upregulated gene. The larger the node, the bigger the degree

**Fig. 5:**
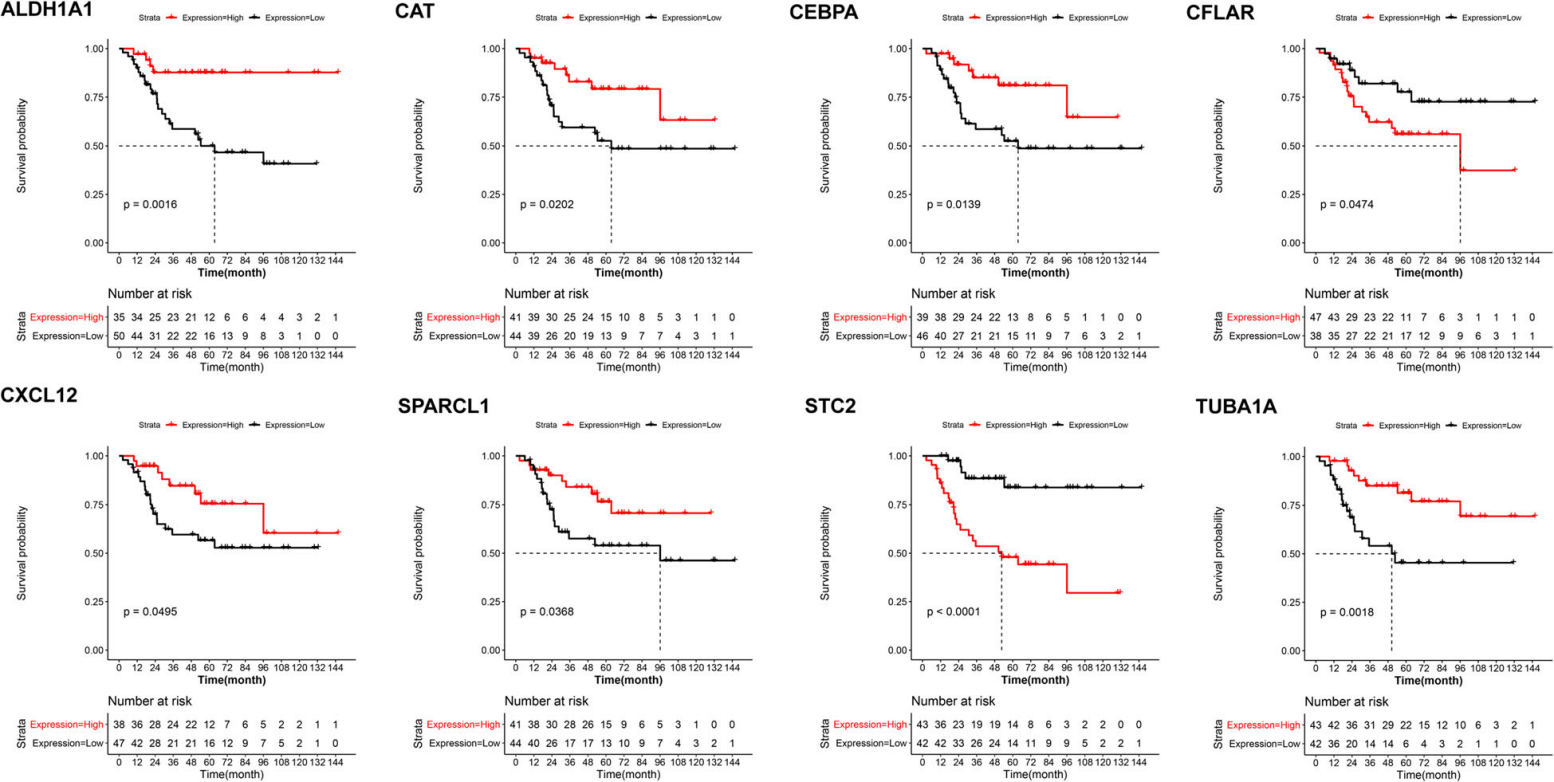
The survival analysis for 8 feature genes. The X-axis represented the survival time (month), while Y-axis represented survival probability

A PPI network was constructed in the current study based on 843 protein interactions and 23 genes. The detailed information for the current PPI network was showed in Fig. 4. Based on genes in the PPI network, CTD database (26355 genes and 3919 compounds) and GeneCards database (26355 genes), the feature genes for OS were explored using VENN plot analysis. The result showed that there were 38 intersected genes (feature genes) in the current study (Figure S2). Then, the survival analysis was performed on all feature genes (Table S1). Finally, a total of 8 feature genes was revealed as prognostic genes. Detailly, expression of genes including CXCL12, CCAAT enhancer-binding protein alpha (CEBPA), SPARC like 1 (SPARCL1), catalase (CAT), tubulin alpha 1a (TUBA1A), and aldehyde dehydrogenase 1 family member A1 (ALDH1A1) were positively correlated with overall survival of patients, while CASP8 and FADD-like apoptosis regulator (CFLAR) and stanniocalcin 2 (STC2) were negatively correlated with overall survival of patients (Fig. 5).

### Verification for prognostic genes expression by qRT-PCR

**Fig. 6:**
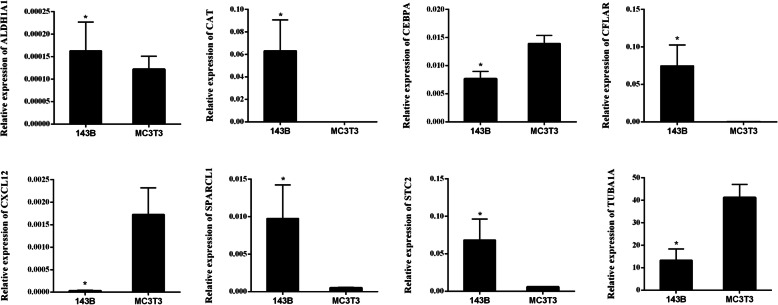
The expression of prognostic genes in tumor cells (143B) and normal cells (MC3T3) detected by qRT-PCR. The X-axis represented different cell lines (groups), while the Y-axis represented the relative expression of different genes. *P < 0.05 when compared with normal

The qRT-PCR was performed to further investigate the expression of 8 prognostic genes in cells (Fig. 6). The results showed that the relative expression of CXCL12, CEBPA, and TUBA1A in tumor cells was significantly lower than that in normal cells (all P < 0.05). Meanwhile, the relative expression of CFLAR and STC2 in tumor cells was significantly higher than that in normal cells (all P < 0.05).

### Disease-metabolic network investigation

As described in methods, a total of 45 compound-gene interactions involving 10 compounds and 8 prognostic genes were obtained from the CTD database (Table S2). Then, using the “Compound ID Conversion” tool provided by MetaboAnalyst database, 5 KEGG metabolite ID corresponding to these 10 compounds were obtained, including C06911, C01692, C13624, C07888, and C01661 (Table S3). These 5 metabolites were OS-associated metabolites.

**Fig. 7:**
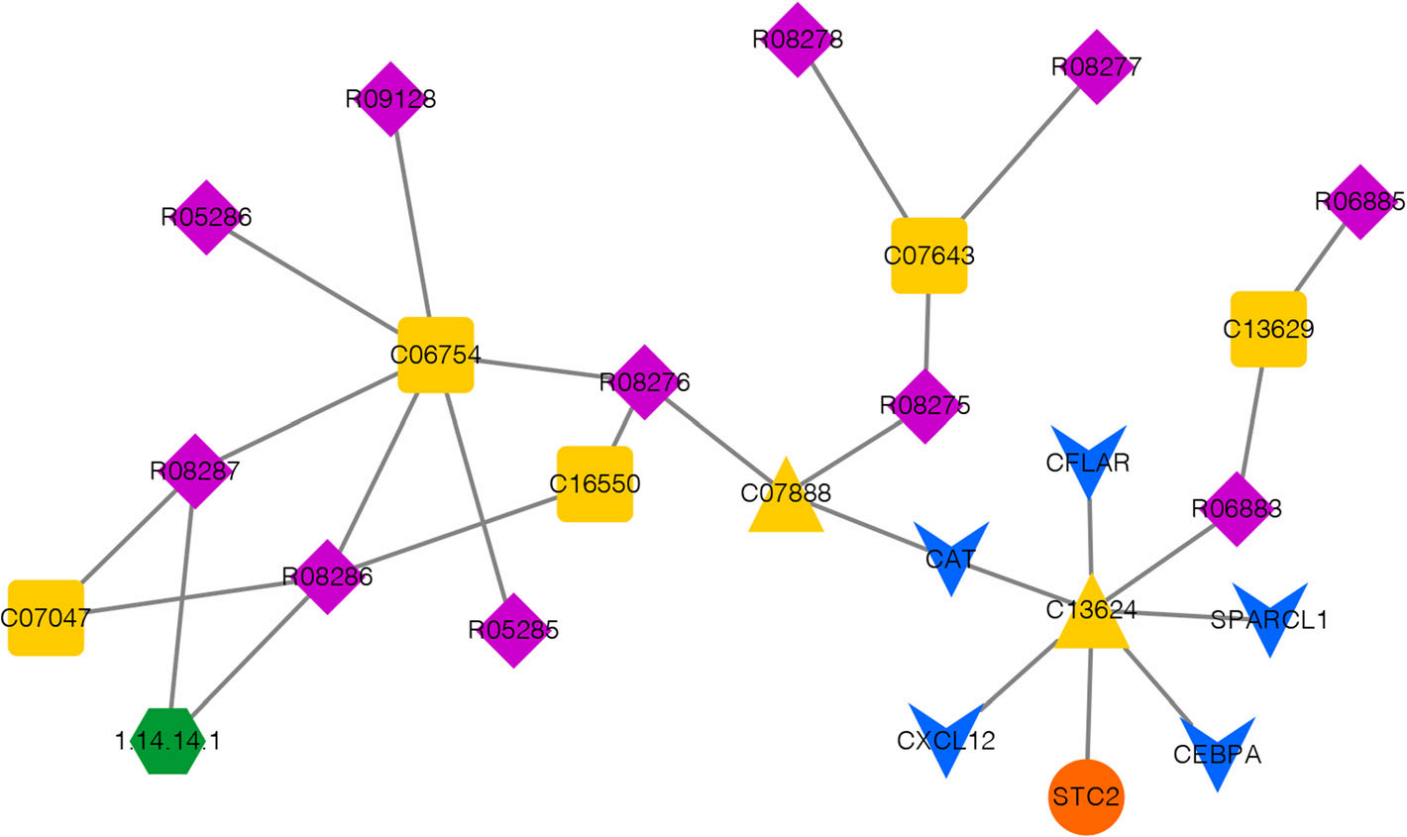
The disease-metabolic network in current study. Green hexagon represented enzyme, pink diamond represented reaction, yellow square represented metabolite, yellow triangle represented disease metabolite, blue inverted triangle represented down regulated gene, and orange circle represented upregulated mRNAs. The line between two nodes represented interaction

Then, a metabolic network was constructed with 10976 nodes and 32242 interactions. Among the 5 OS-associated metabolites, only C13624 and C07888 were matched to the network. With P < 0.05, totally, 19 nodes in the metabolic network were retained, then the DEG-compound interactions were added to the network to generate a disease-metabolic network. The result showed there were 11 reactions, 5 metabolites, 2 disease-metabolites [including C13624 (Bisphenol A, BPA) and C07888 (Cyclophosphamide)], 1 enzyme and 6 DEGs (5 downregulated and 1 upregulated), and 27 interactions in the current disease-metabolic network (Fig. 7, Table 2). Among the disease-metabolite network, C13624 (BPA) showed interactions with all the 6 DEGs was considered as the key OS-associated metabolite.

**Table 2:** Detail information for nodes in current disease-metabolic network

KEGG ID	Entry type	KEGG name	P score
1.14.14.1	Enzyme	Unspecific monooxygenase	4.17E−03
R05285	Reaction	2-Chloroethanol:cytochrome c oxidoreductase	1.00E−06
R05286	Reaction	Chloroacetaldehyde:NAD+ oxidoreductase	1.00E−06
R06883	Reaction	Bisphenol A + NADH + H+ + Oxygen <=> 1,2-Bis(4-hydroxyphenyl)-2-propanol + NAD+ + H_2_O	1.00E−06
R06885	Reaction	1,2-Bis(4-hydroxyphenyl)-2-propanol <=> 4,4'-Dihydroxy-alpha-methylstilbene + H_2_O	1.00E−06
R08275	Reaction	Cyclophosphamide + NADPH + H+ + Oxygen <=> 4-Hydroxycyclophosphamide + NADP+ + H_2_O	1.00E−06
R08276	Reaction	Cyclophosphamide <=> Dechloroethylcyclophosphamide + Chloroacetaldehyde	1.00E−06
R08277	Reaction	4-Hydroxycyclophosphamide <=> 4-Ketocyclophosphamide	3.23E−02
R08278	Reaction	4-Hydroxycyclophosphamide <=> Aldophosphamide	2.08E−03
R08286	Reaction	Ifosfamide <=> Dechloroethylcyclophosphamide + Chloroacetaldehyde	1.00E−06
R08287	Reaction	Ifosfamide <=> 2-Dechloroethylifosfamide + Chloroacetaldehyde	1.54E−05
R09128	Reaction	2-Chloroethanol:cytochrome c oxidoreductase	1.00E−06
C06754	Compound	Chloroacetaldehyde	1.00E−06
C07047	Compound	Ifosfamide	5.24E−05
C07643	Compound	4-Hydroxycyclophosphamide	6.18E−04
C07888	Compound	Cyclophosphamide	1.00E−06
C13624	Compound	Bisphenol A	1.00E−06
C13629	Compound	1,2-Bis(4-hydroxyphenyl)-2-propanol	1.00E−06
C16550	Compound	Dechloroethylcyclophosphamide	1.00E−06

## Discussion

In this study, we screened 357 genes that differentially expressed in human OS cell lines and human normal bone tissue samples. These genes were considered as the key genes involved in the development of OS. Functional enrichment analysis showed that the downregulated genes were mainly enriched in various immune-related functions (such as leukocyte migration and neutrophil activation involved in immune response), while the upregulated genes were mainly enriched in various biosynthetic processes. Similarly, GSVA revealed that various pathways, such as B/T cell receptor signaling pathway, VEGF signaling pathway, cell adhesion molecules pathway, and chemokine signaling pathways showed significant differences between these two groups. The bone shows a highly specialized immune environment, and various immune-related processes and pathways are involved in bone homeostasis. The success of mifamotide, an innate immune stimulator, in adjuvant therapy for non-metastatic OS demonstrates the potential for immune-based therapy to improve the prognosis of patients with OS [24, 25]. Miao et al. suggested that leukocyte recruitment-associated myokines were downregulated in OS, indicating escaping from the host immune system would contribute to the development of OS [26]. Chemokines and their receptors play important roles in the regulation of tumor-mediated immune response in tumors. For example, CXCL1 together with its receptor CXCR2 were implicated in assisting with the homing of neutrophils into the tumor microenvironment in OS [27]. Chemokine receptor CXCR3 expression was found to positively correlated with the abundance of tumor-infiltrating immune cells, such as macrophages M1, CD8 T cells, and activated NK cells in OS [28]. VEGF is an important angiogenesis-promoting factor in various tumors, that has been reported to contribute to the growth and aggressive behavior in OS [29, 30], promote angiogenesis, and inhibit cell apoptosis [31]. Considering these studies, we speculated that the DEGs were involved in the development of OS by regulating pathways associated with immune, chemokines, and VEGF signalings.

In order to screen most valuable DEGs, Venn analysis was performed on genes in the PPI network, and OS-related genes from CTD and Genecards databases, and 38 overlapped genes were identified, among which, 8 genes associated with survival of patients were considered as most valuable genes, including STC2, SPARC1, ALDH1A1, CFLAR, CEBPA, CAT, TUBA1A, and CXCL12. STC2 is a secretory glycoprotein hormone that can regulate cell proliferation and cancer cell lesions [32]. A previous study shows that STC2 is an outstanding immune-related gene during the progression of OS [33]. A recent study indicates that 20 genes including STC2 signatures are identified related to OS, which can be helpful for predicting prognosis of patients with OS [34]. SPARC1 protein can affect osteoblast differentiation, tumorigenesis and tumor metastasis [35]. A previous study indicates that SPARCL1 can block the metastasis of human OS by the upregulation of canonical signaling [36]. As an aldehyde dehydrogenase, ALDH1A1 has been found to be differentially expressed between low and highly metastatic OS [37]. Qi et al. proved that ALDH1A1 was upregulated in the development of OS [38]. In addition, CFLAR is a common therapeutic target in various human cancers including OS [39]. A previous study shows that miR-20a can be used to suppress OS cell proliferation and invasion through CFLAR [40]. CAT encodes catalase, which was found to be involved in regulating the generation of reactive oxygen species, thereby affecting the cytotoxic effects of pimozide on OS cells [41] and regulating apoptosis of p53 null OS MG63 cells [42]. Furthermore, as a potential target in OS treatment, CXCL12 has been proved to participate in the progression and metastasis of bone sarcomas [43]. It is believed that the upregulation of CXCL12 contributes to the positive OS outcome [44]. Actually, the expression of CXCL12 is commonly realized via certain biological functions [45]. Gulino et al. showed that CXCL12 takes part in the variation of mature polymorphonuclear via altered leukocyte response [46]. A previous study indicates that CXCL12 takes part in the trauma and sterile inflammation via leukocyte migration [47]. These studies emphasized the important roles of these genes in the development and progression of OS.

Finally, a disease-metabolic network was constructed including two disease-associated metabolites BPA and cyclophosphamide. Among the disease-metabolites network, C13624 (BPA) showed interactions with all the 6 DEGs were considered as the key OS associated-metabolite. BPA is a widely studied typical endocrine-disrupting chemical [48]. It is closely associated with the clinical treatment of OS [49]. A previous study shows that BPA contributes to the decreasing activity of OS cells and the inhabitation of cell proliferation [50]. Actually, BPA is considered as a prioritized effect biomarker for human biomonitoring [51]. The relationship between BPA and disease risk prediction has already been investigated in a previous study [52]. Our results revealed that BPA interacted with multiple prognosis-related genes, such as CXCL12, STC2, and CFLAR. Thus, we speculated that BPA might be an important metabolite involved in the development of OS by interacting with different genes.

There were some limitations in the current study. (1) The selected GEO dataset was generated from 19 human OS cell lines and 4 human normal bone tissue samples. There might be differences between human tissue samples and cell lines, and differences among different cell lines. In addition, the validation qPCR was performed on the human OS 143B cell line and murine pre-osteoblast MC3T3 cell line. Therefore, a study based on human OS tissue samples and matched adjacent normal samples should be carried out to eliminate the confounding factors. (2) The disease-associated metabolites and metabolite-gene interactions were predicted using online databases. To obtain more reliable results, an integrated analysis based on metabolomics data and transcriptomics should be performed to investigate the differential metabolites and their roles in the development of OS. (3) Eight prognosis-related genes were screened. However, their prognostic value had not been confirmed, and their clinical value should be further evaluated by clinical data.

## Conclusion

In conclusion, eight prognosis-related genes were identified in OS. The downregulated CXCL12 might take part in the progression of OS via participating in the leukocyte migration function. Moreover, mRNAs including CXCL12 and STC2 might be two novel biomarkers for OS. Furthermore, BPA might be an important metabolite interacting with multiple prognosis-related genes in OS.

## Supplementary Information

**Additional file 1: Figure S1.** The workflow of the current study.

**Additional file 2: Figure S2.** The VENN plot analysis for feature genes of osteosarcoma.

**Additional file 3: Table S1.** Survival data and results for survival analysis of all feature genes.

**Additional file 4: Table S2.** The 45 compounds-genes interactions obtained from CTD database.

**Additional file 5: Table S3.** The five KEGG metabolites ID corresponding to these 10 compounds.

## Data Availability

Not applicable. This study was only the primary research, and further study has been in progress.

## Abbreviations

OS: Osteosarcoma
DEGs: Differentially expressed genes
miRNAs: MicroRNAs
GO-BP: Gene ontology-biological process
KEGG: The Kyoto Encyclopedia of Genes and Genomes
STRING: Search Tool for the Retrieval of Interacting Genes
GSVA: Gene set variation analysis
CTD: Comparative toxicogenomics database
UCSC: University of California Santa Cruz
FBS: Fetal bovine serum
α-MEM: α-Minimal essential medium
CXCL12: C–X–C motif chemokine ligand 12
PHGDH: Phosphoglycerate dehydrogenase
FCGR3B: Fc fragment of IgG receptor IIIb
CEBPA: CCAAT enhancer-binding protein alpha
SPARCL1: SPARC like 1
CAT: Catalase
TUBA1A: Tubulin alpha 1a
ALDH1A1: Aldehyde dehydrogenase 1 family member A1
CFLAR: CASP8 and FADD-like apoptosis regulator
STC2: Stanniocalcin 2
VEGF: Vascular endothelial growth factor
BPA: Bisphenol A
