# Supplementary figures and images for: Bioinformatics integrated analysis to investigate candidate biomarkers and associated metabolites in osteosarcoma
Source: J Orthop Surg Res. 2021 Jul 5;16:432. doi: 10.1186/s13018-021-02578-0 (PMC8256509; doi:10.1186/s13018-021-02578-0)

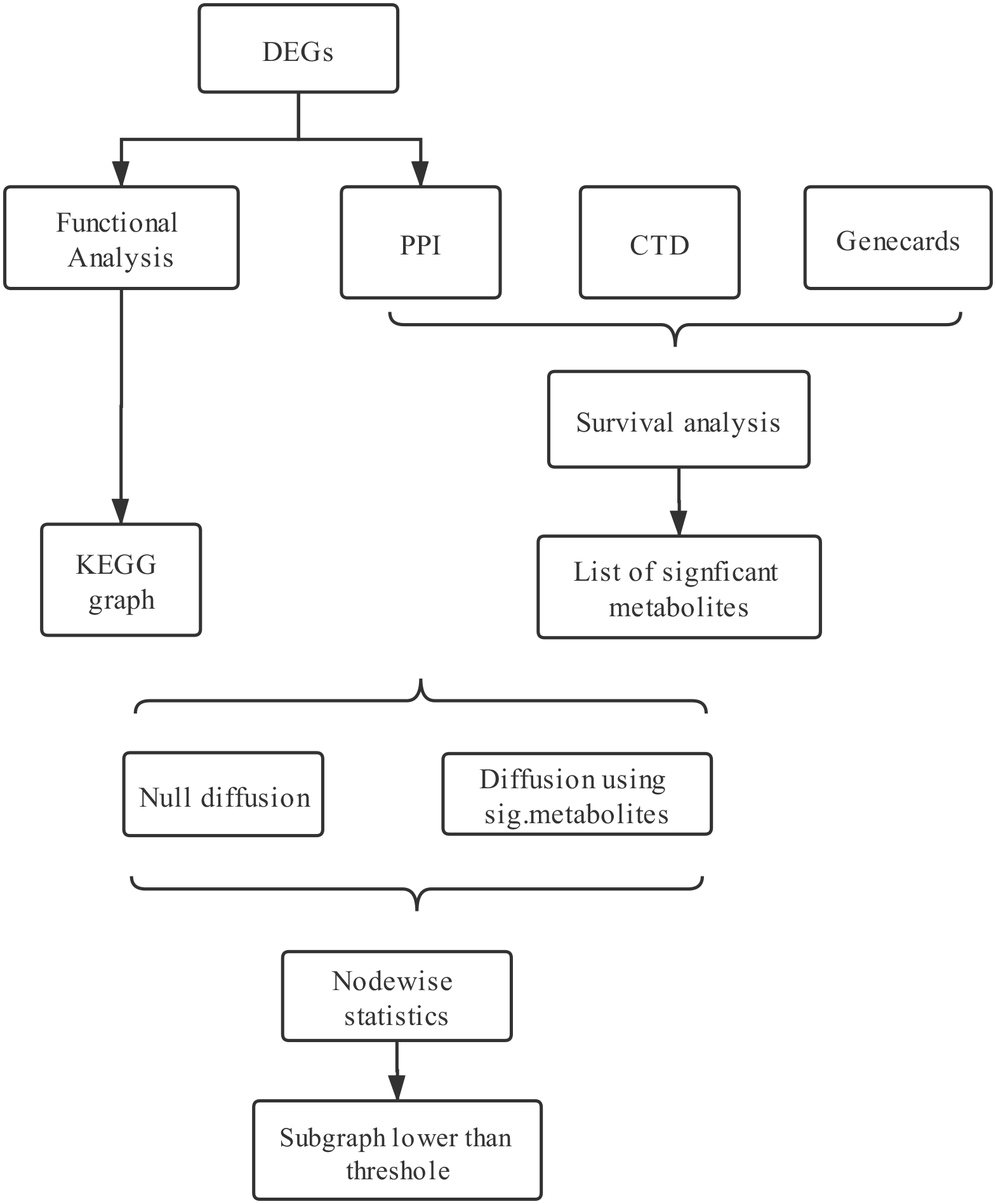

Supplement: Supplementary file 1 — Additional file 1: Figure S1. The workflow of the current study. [file 13018_2021_2578_MOESM1_ESM.tif]

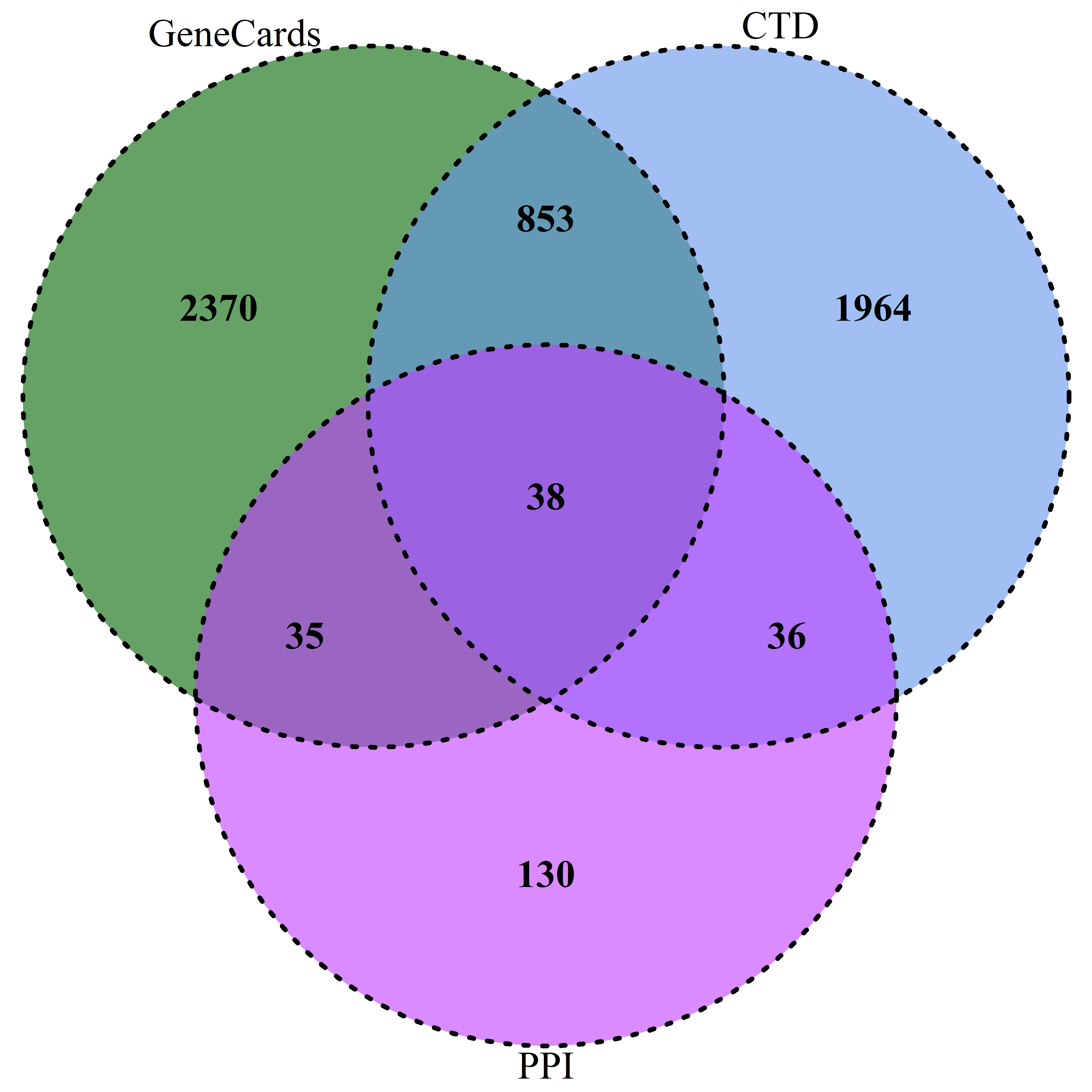

Supplement: Supplementary file 2 — Additional file 2: Figure S2. The VENN plot analysis for feature genes of osteosarcoma. [file 13018_2021_2578_MOESM2_ESM.tif]
